# Cardiac repolarization abnormalities in children with familial Mediterranean fever

**DOI:** 10.1186/s12969-022-00696-5

**Published:** 2022-05-23

**Authors:** Yomna Farag, Shaimaa Sayed, Fatma Alzhraa Mostafa, Huda Marzouk, Raghda H. Mohamed, Rodina Sobhy

**Affiliations:** 1 Pediatrics department, Faculty of Medicine, Cairo University, 4 extension of Nobar Street, Cairo, Egypt; 2 Pediatrics department, National Research Center, Cairo, Egypt

**Keywords:** Arrhythmias, Cardiac repolarization, Familial Mediterranean fever

## Abstract

**Background:**

Familial Mediterranean fever (FMF) is an autoinflammatory disease that can have conduction disturbances and cardiac rhythm disorders as manifestations of cardiac involvement. The aim of the study is to assess the susceptibility of children with FMF to cardiac repolarization abnormalities and therefore arrhythmia in children with FMF.

**Methods:**

A cross sectional study conducted on 60 children had FMF and 40 age and sex matched healthy controls. Cardiac repolarization markers, cardiac dimensions and functions were assessed by electrocardiogram (ECG) and conventional echocardiography in patients and controls.

**Results:**

The mean ± SD age of the patients was 10.43 ± 3.472 years, corrected QT (QTc) and the ratio of peak to end T wave (Tpe) over QTc interval (Tpe /QTc) increased significantly in FMF patients more than healthy control (*p* value 0.023 and 0.022 respectively). P wave dispersion (Pd) was significantly higher in FMF patients with amyloidosis (*p* value 0.030). No significant difference was found in cardiac dimensions and functions between the two groups. We found a statistically negative correlation between Pd and age of patients at time of study, age of disease onset and age at diagnosis. On the other hand, we found a statistically significant positive correlation between Pd with number of attacks per year and disease severity score. Furthermore, Tpe/QTc ratio correlated with FMF 50 score, QTc correlated with 24 hours proteinuria. QT, JT intervals correlated with fibrinogen.

**Conclusions:**

FMF Patients may have increased risk of arrhythmia and should be monitored on regular basis. Compliance to colchicine therapy and better disease control might play a role in decreasing this risk.

## Background

Familial Mediterranean fever (FMF) is the most common autosomal recessive autoinflammatory disease. It is characterized by recurrent, yet self-limiting attacks of serositis in the form of fever, peritonitis, arthritis, pleuritis, and erysipelas-like erythema [1].

FMF is caused by mutations in the MEFV gene, which located on the short arm of chromosome 16 [2]. This gene encodes a protein called pyrin, which is associated with the interleukin-1 inflammatory cascade [3].

Amyloidosis is the most serious complication of FMF as it leads to end stage renal failure and other organ dysfunction [4]. It has been reported in about 13% of FMF patients [5].

These patients are at risk of cardiovascular complications even in the absence of known cardiovascular risk factors [6]. Different cardiovascular manifestations had been reported in FMF as valvular affection, pericarditis and cardiomyopathy [7]. Also, arrhythmia and impairment of both right and left ventricle functions can be observed in patients with FMF [8].

Variations in the duration of the action potential would create cardiac repolarization abnormalities that could be arrhythmogenic and thus be an important predictor of cardiovascular mortality in FMF patients [9].

The risk of cardiac arrhythmias can be predicted from the ECG by measuring several repolarization markers [10]. Some of the most used predictors in clinical practice are: QT interval and its correction by heart rate (QTc), QT interval dispersion (QTd) and other markers like T wave peak to end (Tpe) and these markers are used to predict ventricular arrhythmias [11]. P wave dispersion (Pd) clinical evaluation has been performed in the assessment of the risk for atrial fibrillation [12].

The aim of this work is to assess the susceptibility of children with FMF to cardiac repolarization abnormalities and therefore arrhythmia and sudden cardiac death (SCD).

## Patients and methods

A cross-sectional case control study conducted on 60 children with FMF aiming to assess the susceptibility of children with FMF to cardiac repolarization abnormalities and therefore arrhythmia. Patients were diagnosed according to new FMF criteria in children [13]. All patients, who were included in the study had age of disease onset below 16 years and were in attack-free period for at least 2 weeks. Exclusion criteria included the presence of associated autoimmune disorder, acute or chronic infection, underlying congenital or acquired cardiac diseases, ingestion of drugs that may have an effect on ECG (including non-cardiac drugs causing rhythm disturbance as a side effect) or chronic diseases. Forty apparently healthy children age and gender matched were included as controls. They were coming for regular check-up in the outpatient clinics.

The study had been approval by the ethical committee of Cairo University. After obtaining a written informed consent from the guardians of the children, we collected data that included age of child at time of study, gender, age at disease onset, age at diagnosis, disease duration, number of FMF attacks per month and per year and duration of attack before initiation of colchicine treatment and at time of the study, dose and duration of colchicine. The patients were not receiving anti-IL1 or other medications. We asked about clinical pattern of the disease including fever, abdominal pain, arthritis, erysipelas-like rash, testicular affection and presence of vasculitis. MEFV gene mutations were done using real-time PCR. Measurement of the blood pressure and counting the heart rate were performed. Body mass index (BMI) was calculated. Disease severity was assessed by FMF severity score [14], while response to colchicine treatment was assessed by FMF 50 score [15].

Laboratory tests were done at time of study including; complete blood count (CBC), erytherocyte sedimentation rate (ESR), C-reactive protein (CRP), serum creatinine, sodium, potassium, lipids profile, fibrinogen and 24 hours urinary proteins.

Twelve leads ECG were recorded for all patients and control. The following parameters were measured manually with calibers & a magnifying glass:

1. **QT interval** was measured manually from the point where the Q wave started and the T wave returned to the isoelectric line.
2. **The JT interval** was measured by subtracting the QRS duration from the QT interval.
3. **QT and JT dispersion** were defined as the difference between the longest and shortest QT and JT interval respectively.
4. **Corrected QT (QTc) and corrected JT (JTc)** were performed with the Bazett’s formula [16].
5. **The peak to end interval of T wave (Tpe)** was measured as the distance between the peak and the end of the T wave in the percordial lead V5 (or in leads V4 & V6 if not measurable in V5).
6. **Tpe/ QTc ratio** was calculated from their measurements.
7. **P wave dispersion** (Pd) was calculated as the difference between the longest and shortest P waves.

### Echocardiography

Trans-thoracic two dimensional (M mode) and Doppler echocardiogram were performed. Measurements of left ventricle (LV) cavity was obtained in the form of left ventricle end diastolic diameter (LVEDD), left ventricle end systolic diameter (LVESD), walls (interventricular septum [IVS] and posterior wall). Calculation of fractional shortening (FS %) as an indicator of LV systolic function was done. FS value < 28% considered lower than normal with impaired LV systolic function [17]. The dimensions of the right ventricle (RV) were measured according to recommendations of the American Society of Echocardiography. In addition, left atrial (LA) size and aortic dimension (AO) was determined.

### Statistical methods

Data were statistically described in terms of mean ± standard deviation (±SD), median and range, or frequencies (number of cases) and percentages when appropriate. Comparison of numerical variables between the study groups was done using Student t test for independent samples in comparing 2 groups of normally distributed data and Mann Whitney U test for independent samples for comparing not-normal data. Comparison of normally distributed numerical variables between more than two groups was done using Kruskal Wallis test. For comparing categorical data, Chi-square (X2) test was performed. Exact test was used instead when the expected frequency is less than 5.Correlation between various variables was done using Spearman rank correlation eq. *P* values less than 0.05 was considered statistically significant. All statistical calculations were done using computer program IBM SPSS (Statistical Package for the Social Science; IBM Corp, Armonk, NY, USA) release22 for Microsoft Windows.

## Results

Demographic data, clinical manifestations, laboratory parameters and data about colchicine therapy are shown in (Table 1).

**Table 1:** Demographic data, clinical manifestations and laboratory data of the patients

Parameters	Cases (Mean ± SD)	Control (Mean ± SD)	*P* value
Gender; number (%)			0.935
Male	32 (53.3%)	21 (52.5%)	
Female	28 (46.7%)	19 (47.5%)	
Age (years)	10.43 ± 3.472	12.61 ± 2.32	0.829
Age at onset of disease (years)	4.57 ± 2.197		
Age at diagnosis (years)	6.13 ± 2.771		
Delay in diagnosis (months)	17.33 ± 12.132		
Disease duration (years)	5.86 ± 2.697		
Number of attacks/month	0.62 ± 0.613		
Number of attacks/year before treatment	29.58 ± 16.939		
Number of attacks/year after treatment	6.72 ± 5.149		
Duration of attack (hour) before treatment	52.08 ± 8.796		
Duration of attack (hour) after treatment	19.17 ± 5.221		
Systolic blood pressure ± SD (mmHg)	104.5 ± 10.239	106.25 ± 9.320	0.388
Diastolic blood pressure ± SD (mmHg)	63.25 ± 8.276	64.13 ± 4.789	0.547
Heart rate (beat/minute)	85.48 ± 9.58		
Sodium (mmol/l)	137.62 ± 1.403		
Potassium (mEq/l)	4.282 ± 0.4316		
ESR (mm/hr)	17.65 ± 17.294		
CRP (mg/dl)	5.105 ± 4.8766		
Fibrinogen* (mg/dl)	300.50 ± 65.063		
24 hours proteinuria (mg)	660 ± 170.715		
**Severity score**	**Number (%)**		
Mild disease	13 (21.66%)		
Moderate disease	31 (51.33%)		
Severe disease	16 (26.66%)		
Dose of colchicines (mg)	1.408 ± 0.4738		
Duration of colchicine (years)	4.48 ± 2.555		
**FMF 50 score**	**Number (%)**		
Good responders	58 (96.7%)		
Poor responders	2 (3.3%)		

*SD* Standard deviation, *P* value Probability value, *ESR* Erytherocyte sedimentation rate, *CRP C*-reactive protein, *FMF* Familial Mediterranean fever

This study included 60 patients with FMF; 32 (53.3%) were males and 28 (46.7%) females. Mean ± SD age of the patients was 10.43 ± 3.472 years. The control group included 40 age and gender matched healthy children, 21 (52.5%) were males and 19 (47.5%) females. Their mean age was 12.61 ± 2.32 years.

Patients had a mean age of diagnosis 6.13 ± 2.771 years. Positive family history of FMF was found in 36.7% of patients. The number of FMF attacks per year at disease diagnosis were 29.58 ± 16.939 (range: 3–60) and decreased to 6.72 ± 5.149 (range: 0–20) at time of study.

Systolic and diastolic blood pressures and BMI were within normal range and no statistically significant difference between cases and controls (Table 1. FMF manifestations during the attacks were fever, abdominal pain were in all patients (60/100%) followed by arthritis (29/48.3%). Nearly half the patients had moderated disease severity (31/51%). Most of the patients (58/96.7%) were good responders to colchicine therapy who had 50% improvement of the 6 criteria of the FMF 50 score [15]. The dose of colchicine was 1.408 ± 0.4738 mg. Ten patients (16.66%) had long QTc ranging between 464.1 and 482.2 msec.

Valvular involvement was detected by echocardiography in (10/16.66%) of patients in the form of mild MR (4/6.66%), trivial MR (5/8.33%), and mild AR (1/1.7%). In the control group, valvular involvement was detected in one child in the form of mild MR without statically significant difference between the patients and control group. Also, there was no statically significant difference between valvular involvement and Pd or QTc changes.

There was no statistically significant difference between patients and controls regarding echocardiographic diameters and contractility. Fraction shortening and ejection fraction were close in patients and control (38.35 ± 4.157 versus 39.83 ± 4.132%, *p* value 0.085 and 68.82 ± 5.357 vs 69.33 ± 4.190%, *p* value 0.597 respectively) (Table 2). On the other hand, Tpe and Pd were longer in the patients with no statistical significance (*p* value 0.07 and 0.135 respectively). Furthermore, statistically significant longer QTc, JTc and Tpe/QTc ratio were found in cases compared with controls (*p* value 0.023, 0.054 and 0.022 respectively) (Table 2. On the other hand, we didn’t find significant differences in QT, JT intervals, corrected JT (JTc), QT interval dispersion (QTd), JT dispersion (JTd) and peak to end T wave (Tpe) between patients and control.

**Table 2:** Comparison between echocardiography measurements and ECG parameters of patients and controls

Parameter	Patients; mean ± SD	Control; mean ± SD	*P* value
IVST (cm)	0.633 ± 0.0951	0.638 ± 0.0952	0.831
PWT (cm)	0.623 ± 0.1047	0.630 ± 0.1043	0.755
LVEDD (cm)	3.747 ± 0.4515	3.760 ± 0.4662	0.888
LVESD (cm)	2.453 ± 0.4616	2.390 ± 0.3754	0.453
FS (%)	38.35 ± 4.157	39.83 ± 4.132	0.085
EF (%)	68.82 ± 5.357	69.33 ± 4.190	0.597
SV (ml)	49.80 ± 15.578	51.53 ± 15.066	0.581
AO (cm)	1.97 ± 0.281	2.02 ± 0.237	0.349
LA (cm)	2.117 ± 0.2663	2.070 ± 0.2301	0.362
PA (cm)	1.850 ± 0.1321	1.878 ± 0.1000	0.240
RV (cm)	1.773 ± 0.2106	1.775 ± 0.1660	0.965
QT (msec)	371.67 ± 32.634	373 ± 29.544	0.833
JT (msec)	285.67 ± 33.667	288.25 ± 24.692	0.659
QTd (msec)	29.17 ± 14.414	28.50 ± 9.487	0.781
JTd (msec)	25.5 ± 13.074	24.50 ± 8.458	0.643
Tpe (msec)	94.5 ± 11.536	89.50 ± 15.777	0.071
Pd (msec)	20.17 ± 10.969	17.50 ± 6.699	0.135
QTc (msec)	438.38 ± 24.409	427.78 ± 20.966	0.023
JTc (msec)	352.38 ± 25.740	342.78 ± 22.994	0.054
Tpe/QTc ratio	0.2213 ± 0.03237	0.2046 ± 0.03889	0.022

*IVST* Inter ventricular septum thickness, *PWT* Posterior wall thickness, *LVEDD* Left ventricle end diastolic diameter, *LVESD* Left ventricle end systolic diameter, *FS* Fractional shortening, *EF* Ejection fraction, *SV* Stroke volume, *AO* Aorta, *LA* Left atrium, *PA* Pulmonary artery, *RV* Right ventricle, *QTd* QT interval dispersion, *JTd* JT interval dispersion, *Tpe* Peak to end T wave, *Pd* P wave dispersion, *QTc* Corrected QT interval, *JTc* Corrected JT interval, *SD* standard deviation, *P* value Probability value

Patients were divided into two groups according to the presence of amyloidosis. Amyloidosis was considered in 3 patients with persistent proteinuria, but unfortunately the parents of these patients refused to perform renal biopsy for their children. Pd was significantly higher in patients with amyloidosis (*p* value 0.03), while the rest of ECG parameters didn’t show any difference between the two groups (Table 3).

**Table 3:** Comparison between ECG parameters of patients with and without amyloidosis

Parameters	Patients without amyloidosis (N:57) mean ± SD	Patients with amyloidosis (N:3) mean ± SD	*P* value
QT (msec)	372.8 ± 31.002	350 ± 17.321	0.073
JT (msec)	286.4 ± 31.983	270 ± 17.321	0.072
QTd (msec)	29.3 ± 13.693	26.67 ± 11.547	0.483
JTd (msec)	25.4 ± 12.420	26.67 ± 11.547	0.863
QTc (msec)	439.08 ± 23.188	425 ± 24.754	0.891
JTc (msec)	355.77 ± 24.453	344.97 ± 24.803	0.459
Tpe (msec)	94.73 ± 10.959	90 ± 10	0.467
Tpe/QTc ratio	0.2054 ± 0.0369	0.189 ± 0.0517	0.411
Pd (msec)	19.654 ± 10.420	26.67 ± 11.547	0.030

QTd: QT interval dispersion, JTd: JT interval dispersion, QTc: corrected QT interval, JTc: corrected JT interval, Tpe: peak to end T wave, Pd: P wave dispersion, N: number, SD: standard deviation, *p-*value: probability value

Fifteen patients (25%) had borderline QTc ranging between 441.1 and 457.4 milliseconds; mean ± SD age was 10.86 ± 4.15 years.

We presented the correlation between different ECG parameters of the patients with their demographic data, clinical manifestations, disease severity score and colchicine therapy (Tables 4 and 5). We found positive correlation between QT and JT with the age at time of study (*p-*value 0.022, 0.014 respectively), disease duration (*p-*value < 0.001) and duration of attack in hours at time of study (*p-*value 0.042, 0.012 respectively). Pd correlated negatively with the child’s age at time of study (*p-*value 0.007), at disease onset (*p-*value 0.018) and at diagnosis (*p-*value 0.019). On the other hand, it correlated positively with number of attacks per month (*p-*value 0.022) and per year at time of study (*p-*value 0.035), erysipelas like erythema (*p-*value 0.010) and severity score (*p-*value 0.05).

**Table 4:** Correlation between ECG parameters of the patients with their history parameters and colchicines data

History parameters		QT	JT	QTc	JTc	Pd
Age at study	Coefficient correlation	0.296	0.314	−0.007	0.025	− 0.306
	*P* value	0.022*	0.014	0.960	0.852	0.018*
Age disease onset	Coefficient correlation	−0.127	−0.106	−0.057	− 0.035	−0.343
	*P* value	0.335	0.422	0.666	0.793	0.007*
Age of diagnosis	Coefficient correlation	−0.068	−0.040	− 0.107	− 0.080	−0.303
	*P* value	0.607	0.760	0.417	0.542	0.019*
Disease duration	Coefficient correlation	0.453	0.450	−0.054	−0.016	−0.108
	*P* value	0.000*	0.000*	0.682	0.905	0.409
Number of attack/month	Coefficient correlation	0.084	−0.053	−0.048	− 0.184	0.295
	*P* value	0.522	0.687	0.714	0.158	0.022*
Number of attack/year at time of study	Coefficient correlation	0.038	−0.096	−0.105	−0.187	0.273
	*P* value	0.773	0.466	0.423	0.152	0.035*
Duration of attack at time of study	Coefficient correlation	−0.264	−0.322	−0.077	− 0.150	0.238
	*P* value	0.042*	0.012*	0.559	0.252	0.067
Erysipleas like erythema	Coefficient correlation	0.067	0.187	0.163	0.302	0.329
	*P* value	0.610	0.153	0.213	0.019*	0.010*
Severity score	Coefficient correlation	0.088	0.040	0.090	0.088	0.255
	*P* value	0.506	0.759	0.492	0.502	0.050*
Colchicine data		**QT**	**JT**	**QTd**	**Tpe**	**Tpe/QTc**
Dose of colchicine	Coefficient correlation	0.229	0.284	−0.005	0.021	−0.012
	*P* value	0.079	0.028*	0.970	0.875	0.928
Duration of colchicine treatment	Coefficient correlation	0.440	0.446	0.124	0.168	0.145
	*P* value	0.000*	0.000*	0.347	0.201	0.207
FMF50 score	Coefficient correlation	−0.106	−0.100	0.145	0.224	0.271
	*P* value	0.419	0.446	0.270	0.085	0.036*

*QTc* Corrected QT interval, *JTc* Corrected JT interval, *Pd* P wave dispersion, *QTd* QT interval dispersion, *Tpe* Peak to end T wave, *P* value Probability value

**Table 5:** Correlation between ECG parameters of the patients and their investigations

parameters		QT	JT	QTd	QTc	JTc	Tpe/QTc	Pd
Sodium	Coefficient correlation	0.235	0.192	0.077	−0.283	−0.238	0.242	−0.138
	*P* value	0.070	0.141	0.558	0.028*	0.067	0.062	0.294
Urea	Coefficient correlation	−0.090	−0.166	−0.131	− 0.262	−0.267	0.136	0.017
	*P* value	0.493	0.205	0.317	0.044*	0.039*	0.299	0.899
Fibrinogen	Coefficient correlation	0.364	0.391	0.126	−0.250	−0.240	0.020	0.003
	*P* value	0.004*	0.002*	0.339	0.054	0.064	0.882	0.984
24 hours proteinuria	Coefficient correlation	0.054	0.095	0.102	0.268	−0.205	0.218	0.160
	*P* value	0.680	0.469	0.439	0.039*	0.116	0.094	0.221

*QTd* QT interval dispersion, *QTc* Corrected QT interval, *JTc* Corrected JT interval, *Tpe* Peak to end T wave, *Pd* P wave dispersion, *P* value Probability value

Dose of colchicine therapy at time of study correlated with JT interval (*p-*value 0.028). Furthermore, JT interval, together with QT intervals, correlated with duration of colchicine treatment (*p-*value 0.00). Furthermore, Tpe/QTc ratio correlated with FMF50 score (*p-*value 0.036) (Table 4).

There was positive correlation between QTc interval and 24 hours proteinuria (*p-*value 0.039). Furthermore, QT and JT intervals correlated with fibrinogen (*p-*value 0.004 and 0.002 respectively) (Table 4).

## Discussion

The current study found prolonged QTc and increased Tpe/QTc ratio in children with FMF compared to their healthy control, which might increase their risk of having ventricular arrythmias. Furthermore, P wave dispersion (Pd) increased significantly in FMF children with amyloidosis putting them at higher risk for atrial arrythmia. This finding might be related to the systemic inflammation associated which FMF that can affect the heart as well.

Furthermore, we didn’t find a significant difference in QT, JT intervals, corrected JT (JTc), QT interval dispersion (QTd), JT dispersion (JTd) and peak to end T wave (Tpe). This finding was in concordance with other studies who found that children with FMF and the control group had significant difference in QTc [16, 18, 19]**,** and similar QT, JT, JTc, QTd and JTd [16, 19]**.**

QTc value is an important marker of cardiovascular mortality as it indicates the increased ventricular sensitivity. It has an important effect on sudden cardiac death and arrhythmia. So, ECG monitoring may be useful in patients with FMF [20]**.**

On the other hand, Israeli study conducted by Nussinuvich et al. 2011 didn’t find a higher risk for ventricular arrythmias in FMF patients, even in those having amyloidosis [21]**.** This discrepancy might be related to the effect of ethnicity on the genetic mutations and the severity of the disease, causing different disease phenotypes among different populations.

P wave dispersion (Pd), which is a marker for potential atrial arrythmias, showed no statistically significant difference between cases and control group in the current study. This finding matched other studies [12, 21]**.** This finding might be explained by the effect of colchicine treatment on decreasing inflammation and its adverse effects on different body systems, including the heart.

In the current study, there was no significant difference in cardiac dimensions and systolic functions between patients and controls. This finding matched other studies [19, 22]**.** Valvular involvement was detected by Echocardiography in (10/16.66%) of patients. Similar valvular abnormalities were reported in FMF patients by Salah et al [23].

We found a positive correlation between QT, JT intervals and the age of patients at time of study, duration of disease, FMF attacks and colchicine treatment. These two intervals correlated with serum fibrinogen level as well.

P wave dispersion (Pd) had a positive correlation with the frequency of FMF attacks and FMF severity score. This means that children with severe FMF disease might have increased risk for atrial fibrillation. Furthermore, significant negative correlation was found between Pd and age of patients at study time, their age at disease onset, and at diagnosis.

We found a positive correlation between JT interval and dose of colchicine therapy. This agrees with Nussinovich et al. 2020 [24] who found that FMF patients under colchicine therapy might have a lower risk for cardiac arrhythmias when compared to newly diagnosed FMF patients. So, we think that compliance to colchicine therapy and better control of the disease might have a protective role in decreasing risk of arrhythmias in these patients.

Another significant positive correlation was found between QTc and 24 hours proteinuria; which indicate that increased 24 hours proteinuria as a part of FMF disease progression carries the risk of prolongation in QTc, repolarization abnormalities and ventricular arrhythmia.

The current study showed that FMF had cardiovascular morbidity in the form of ECG changes and cardiac valvular abnormalities in spite of there was no symptomatic arrhythmia or mortality.

### Study limitations

Some of the limitations of this study includes being a cross sectional study. We hope that patients would be followed prospectively for arrhythmic episodes in further studies.

## Conclusion

We concluded that children with FMF are susceptible for arrhythmia and sudden cardiac death due to cardiac repolarization abnormalities. Prolonged QTc and increased Tpe/QTc ratio are common finding in FMF patients. FMF children with amyloidosis may be at a higher risk for arrhythmia especially atrial arrhythmia and fibrillation. Regular ECG recording and echocardiographic evaluations of cardiac functions for FMF children are mandatory. Compliance to colchicine therapy and better control of the disease might have a role in decreasing risk of arrhythmias in these patients.

## Data Availability

The datasets used and/or analyzed during the current study are available from the corresponding author on reasonable request.

## Abbreviations

AO: Aorta
EF: Ejection fraction
CBC: Complete blood count
CRP: C-reactive protein
ESR: Erytherocyte sedimentation rate
FMF: Familial Mediterranean fever
FS: Fractional shortening
IVST: Inter ventricular septum thickness
JTc: Corrected JT interval
JTd: JT interval dispersion
LA: Left atrium
LV: Left ventricle
LVEDD: Left ventricle end diastolic diameter
LVESD: Left ventricle end systolic diameter
PA: Pulmonary artery
Pd: P wave dispersion
*P* value: Probability value
PWT: Posterior wall thickness
QTc: Corrected QT interval
QTd: QT interval dispersion
RV: Right ventricle
SCD: Sudden cardiac death
SD: Standard deviation
SV: Stroke volume
Tpe: Peak to end T wave
